# The Potential Role of the Bacterial Persister Formation Gene ptsH in Hypervirulence Development in Klebsiella pneumoniae

**DOI:** 10.32607/actanaturae.27800

**Published:** 2026

**Authors:** A. V. Tutel’yan, N. V. Vlasenko, V. M. Pisarev, Yu. V. Mikhailova, N. V. Sycheva, A. A. Tarlycheva, A. A. Shelenkov, D. K. Kondrat’eva, V. G. Akimkin

**Affiliations:** Central Research Institute of Epidemiology, Federal Service for the Oversight of Consumer Protection and Welfare (Rospotrebnadzor), Moscow, 111123 Russia; Federal Research and Clinical Center of Intensive Care Medicine and Rehabilitology, Ministry of Science and Higher Education of the Russian Federation, Moscow, 107031 Russia

**Keywords:** K. pneumoniae, hypervirulence, persister formation, PTS system, ptsH gene, aerobactin, sequence typing

## Abstract

Persistence – i.e., the ability to exist in a metabolically inactive form – allows bacteria to accumulate genetic advantages. The evolution of the pathogenic potential of Klebsiella pneumoniae has led to the emergence of strains simultaneously characterized by increased aggressiveness (virulence) and prolonged survival in the host organism. This combination of properties contributes to the emergence of “superbugs,” necessitating the search for specific markers that would make it possible to prevent the spread of highly adaptive clones. Hypervirulent K. pneumoniae (hvKp) strains represent a growing global health threat, since they combine high invasiveness and antibiotic resistance. An analysis of 92 K. pneumoniae clinical isolates was conducted to assess the prevalence of the key hypervirulence genes (iroB, peg-344, rmpA, rmpA2, and iucA) and investigate their association with the bacterial persister formation gene ptsH. It was found that 64.1% (59/92) of the isolates carried at least one hvKp gene, iucA being the most frequent one (62.0%). The full set of five hvKp genes was identified in only one case (1%). The strains of sequence types ST23, ST268, ST86, ST534, ST219, ST101, and ST395 accumulated virulence genes, whereas ST512 and ST14 rarely harbored hvKp genes. A key finding was the detection of a significant association between the presence of the ptsH gene (found in 50% of the strains) and the accumulation of hvKp genes: the ptsH-positive strains were statistically more likely to harbor the complete aerobactin operon (iucABCD), in combination with one or more additional hypervirulence genes, compared to the ptsH-negative strains (p < 0.05). Our findings indicate that the ptsH gene is crucial in the formation of polygenically determined hypervirulence, and that its role in controlling bacterial persistence creates evolutionary advantages under stress induced by antibiotics or immune factors, thus promoting evasion of their actions. The phosphotransferase system (PTS), to which the ptsH gene belongs, can potentially become a novel source of molecular targets for the therapy of infections caused by hypervirulent K. pneumoniae strains.

## INTRODUCTION

Persistence is defined as the ability of a microorganism to enter a non-replicating, metabolically quiescent state and survive in this form over extended periods of time. This capacity provides bacterial populations an ecological niche facilitating the preservation and accumulation of genetic determinants, which may, under certain conditions, confer selective advantages to them. Persister cell formation is also a feature of Klebsiella pneumoniae, a bacterial species whose pathogenic potential has undergone substantial evolution, having transformed from a classical opportunistic pathogen to a global health threat [1]. The combination of traits that simultaneously enhance aggressiveness and long-term survival within a macroorganism promotes the emergence of the so-called “superbug.”

K. pneumoniae has long been viewed as a classic opportunistic pathogen, primarily associated with nosocomial infections in healthcare settings. However, in the late 20th and early 21^st^ centuries, severe community-acquired K. pneumoniae infections began to emerge in immunocompetent individuals [2, 3, 4, 5, 6, 7]. This phenomenon has been attributed to the appearance of hypervirulent K. pneumoniae (hvKp) strains, which are now widely distributed globally [8]. Hypervirulence is defined as enhanced functional activity of a bacterial strain toward the human host, driven by activation of a complex array of bacterial genetic determinants. Hypervirulent K. pneumoniae (hvKp) strains are characterized by a specific set of genetic determinants. It is believed that the full deployment of hypervirulence requires the concurrent presence of five key genes [9], two of which (iucA and iroB) belong to clusters involved in bacterial siderophore biosynthesis. The iucA gene is a component of the aerobactin operon (iucABCD) and a reliable marker for the presence of the entire operon, whereas iroB is a component of the salmochelin (enterobactin) operon [10]. The other two genes, rmpA and rmpA2, fulfill a regulatory function by controlling the hypermucoid phenotype formation in the bacterial cell [11]. Finally, this set includes the peg-344 gene coding for the metabolite transport system on the inner membrane of the microorganism [12, 13]. Using a murine model of sepsis [14], together with comprehensive virulence profiling based on several pathogenetically relevant criteria, including the induction of the pathological state in mice via subcutaneous inoculation of different K. pneumoniae strains, as well as by evaluating siderophore production and the mucoid phenotype, it was proved that the concurrent presence of the five genes iucA, iroB, peg-344, rmpA, and rmpA2 allows us to characterize the K. pneumoniae strain as hypervirulent. The presence of all five biomarkers, iucA, iroB, peg-344, rmpA, and rmpA2, came with the highest accuracy (94%), whereas the presence of at least four of them provided the greatest sensitivity (100%) to the method [15]. From the perspective of functional competitive advantages, this genetic repertoire makes hvKp strains capable of efficient iron scavenging under iron-limiting conditions, formation of a protective mucous capsule, and optimized intracellular nutrient transport.

The acquisition and stable maintenance of functionally significant genes within the genome is a crucial aspect of the evolution of bacterial pathogens. Thus, accumulation of plasmid-borne genes enhancing virulence [16] enables K. pneumoniae to gain a competitive advantage over wild-type strains [17]. Of particular interest is the so-called “plasmid paradox” [18], which refers to the stabilization of plasmids carrying hypervirulence genes within a strain, although there is no direct energetic benefit associated with their maintenance. Numerous studies and the diverse hypotheses about the mechanism of this phenomenon, including plasmid–bacterial co-adaptation, enhanced horizontal gene transfer (including cross-species intergeneric transmission), mobile genetic elements facilitating optimal adaptation, and optimization of the transfer of pathogenicity factors, emphasize that this significant issue remains unresolved [19, 20, 21].

Dewar et al. demonstrated that in pathogenic broad-host-range bacteria, plasmids were more likely to carry virulence-associated genes [22]. These findings are consistent with the horizontal transfer of hvKp genes in K. pneumoniae [16, 23], which are also widely distributed among mammals, including animals that are in contact with humans: dogs, cats, and livestock.

The global prevalence of hvKp genes among both nosocomial and community-acquired K. pneumoniae strains is high and tending to rise and is associated with multidrug resistance [24, 25, 26, 27, 28]. This trajectory makes the search for novel strategies to combat aggressive strains more pressing [29, 30]. Yet, the mechanisms underlying the development of hypervirulence – which requires the full complement of five genes, iucA, iroB, peg-344, rmpA, and rmpA2 to be fully manifested – remain poorly understood. We hypothesize that under stress and antibiotic exposure, the plasmids carrying these genes may preferentially accumulate in bacteria characterized by low metabolic activity, high antibiotic tolerance, and immune evasion capabilities. Such criteria are met by bacteria in the persistent state: antibiotic-tolerant, non-replicating bacteria that are optimally adapted to withstand stress factors, including immune defense and antibiotic pressure [3, 6, 29, 30, 31, 32]. These non-replicating yet viable persister bacteria, which survive despite lifethreatening stress conditions (antibiotics, adaptive and innate immune factors, and pH fluctuations), could maximize those benefits by exiting the persister state through expression of the hvKp genes. Therefore, it seems fair to expect that hvKp genes will accumulate in strains concurrently with the genes governing persister formation. Under this scenario, the hypervirulence resulting from the accumulation of hvKp genes in a single strain would be coupled with a genetically determined enhanced capacity for persister formation.

Among the genes associated with the properties typical of bacterial persistence, the ptsH gene encoding the histidine-containing phosphocarrier protein (HPr) of the phosphotransferase system (PTS) has been studied the best. The PTS products are involved in carbohydrate metabolism, stress responses, and interactions with transcription factors. A recent study has proved that the ptsH gene plays a crucial role in the generation of persister forms of K. pneumoniae upon exposure to the levofloxacin antibiotic [33]. Transcription of the ptsH gene was found to be activated during persister formation and suppressed upon resumption of bacterial growth. Using CRISPR-Cas9 to generate a K. pneumoniae ΔptsH knockout strain, it was revealed that ptsH promoted persister formation, reduced the accumulation of reactive oxygen species and enhanced antioxidant activity via downregulating of the cAMP levels. The reduced metabolic activity characteristic of the persistent state can possibly indirectly contribute to the increased plasmid stability by alleviating competition for replication-associated resources, thereby enhancing the likelihood of plasmid retention in bacterial persisters. It seems safe to expect that following the removal of stress and the resolution of the persistence period, ptsH-carrying bacteria that have survived stress exposure would be capable of outcompeting other bacteria, provided that they have acquired the hvKp genes conferring enhanced adaptation of bacteria to host invasion.

In the present study, we searched for a potential association between the ptsH gene and the accumulation of hvKp genes among clinical K. pneumoniae strains responsible for severe nosocomial and community-acquired infections.

## EXPERIMENTAL

To test our hypothesis, we examined bacterial culture samples obtained by the Reference Center for Monitoring of Healthcare-Associated Infections (HAIs) during a period from 2022 to 2023. DNA was extracted using a RIBO-prep reagent kit (Central Research Institute of Epidemiology, Russia). DNA samples for subsequent high-throughput sequencing were prepared using the Illumina Nextera DNA Library Prep Kit and the Illumina Nextera Index Kit (Illumina, USA). Sequencing was carried out on an Illumina NextSeq2000 platform (Illumina). Genome assemblies were generated from the short-read sequencing data using the SPAdes software version 3.15.4 [34] on default parameters. Quality assessment and filtering of the resulting assemblies, verification of bacterial species identification against the initial data, and genome annotation were performed using a bioinformatics pipeline developed previously [35, 36]. Isolate typing was conducted based on multilocus sequence typing (MLST) using the BIGSdb database [37] (accessed May 20, 2024).

Antimicrobial resistance genes were identified using the ResFinder database version 4.5.0 [38] (accessed May 20, 2024); the virulence factors were identified using the Virulence Factor Database (VFDB) [39] (accessed May 20, 2024). Plasmid contig detection and typing within the genomes were performed using the MOB-suite software package [40] on default parameters (mob_typer version 3.1.2).

The study included samples obtained from patients older than 18 years (n = 92) admitted to the intensive care units of medical facilities in Moscow, Russia. Statistical analysis was carried out using the StatTech v. 4.8.5 software (StatTech OJSC, Russia). Differences were considered statistically significant at p < 0.05.

## RESULTS AND DISCUSSION

The prevalence of individual hypervirulence genes among the isolates (irrespective of co-occurrence with other hvKp genes) was as follows: iroB was detected in 5 (5.4%) isolates; peg-344, in 10 (10.9%) isolates; rmpA, in 12 (13.0%) isolates; rmpA2, in 24 (26.1%) isolates; and iucA, in 57 isolates (62.0%). At least one hypervirulence gene was found in 59 samples (64.1%), which was indicative of the high overall prevalence of hvKp-associated genes within the study cohort. Meanwhile, Samoilova et al. [27] reported substantially higher detection rates of hypervirulence genes in their cohort of K. pneumoniae isolates. Thus, rmpA and rmpA2 were identified in 49.3% and 58.7% of isolates, respectively; the iuc and iro loci were detected in 73.3% and 14.7%, respectively

A critical parameter for assessing the virulence of hvKp isolates is the number of hypervirulence genes accumulated per isolate. In our cohort, two and/or more hypervirulence genes were identified in 26 (28.9%) isolates; among these, combinations of any two hvKp genes were observed in twelve isolates; combinations of three genes, in six isolates; and four genes, in seven isolates. The concurrent presence of all five hypervirulence genes was detected in only one K. pneumoniae isolate (the capsular serotype K1, international sequence type ST23, which is characteristic of hvKp strains found among healthcare-associated infections).

**Fig. 1:**
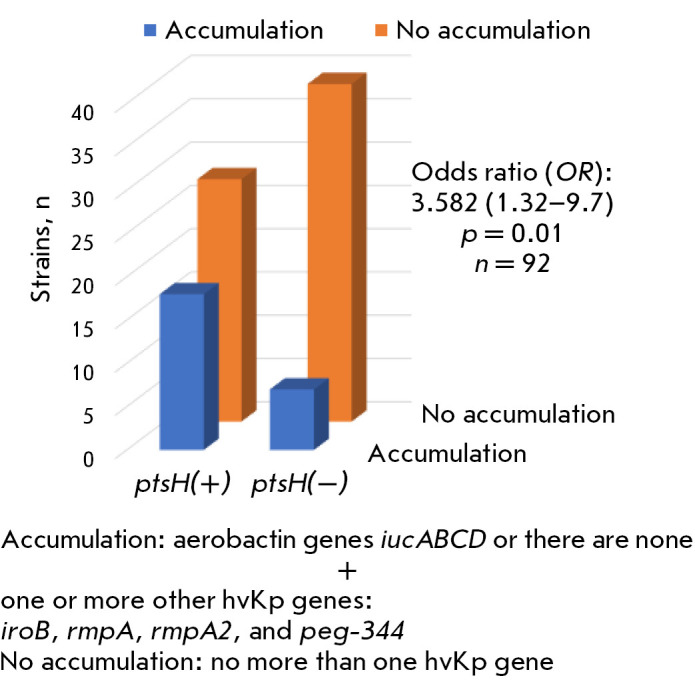
Contribution of the ptsH gene to the accumulation of hypervirulence genes

The ptsH gene, which is implicated in the stabilization of plasmid-borne hypervirulence genes and is a key determinant of the formation of bacterial persister populations [33], was identified in 46 (50%) of the 92 strains examined. Among ptsH-positive isolates, the complete iucABCD operon, in combination with at least one pLVK plasmid-derived hypervirulence gene (iroB, rmpA, rmpA2, and peg-344), was detected in 18 cases. Statistical analysis revealed a significant association between the ptsH gene and the concurrent presence of the complete iucABCD operon, in combination with at least one additional hypervirulence gene (iroB, rmpA, rmpA2, and peg-344) (Fig. 1).

**Fig. 2:**
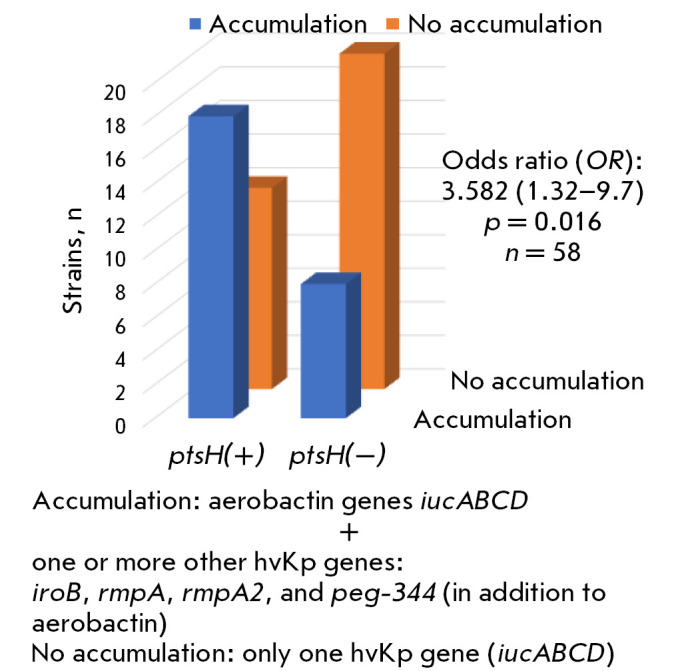
Additional accumulation of hypervirulence genes

The association between the ptsH gene and hvKp genes reached statistical significance in the subgroup of patients under 70 years of age (n = 70), with the odds ratio of 3.74 (95% CI 1.135–12.327; p = 0.043). No such association was observed in patients over 70 years of age, likely because of the limited statistical power of the sample (p = 0.7; n = 22) (Fig. 2).

**Fig. 3:**
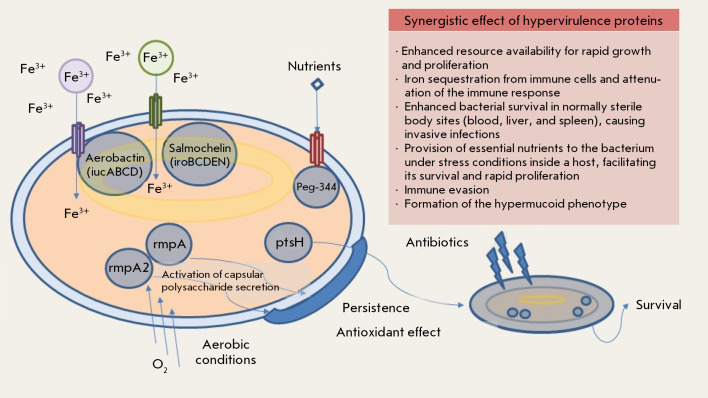
The interplay between hvKp-encoded proteins and the ptsH gene coding for the bacterial persister formation

The identified association between the gene encoding bacterial persister formation and hypervirulence genes can be attributed to two mechanisms. On the one hand, plasmid replication and segregation are ATP-dependent processes. Mutations in and/or the absence of the ptsH gene impair carbohydrate transport, leading to reduced ATP synthesis and destabilization of plasmids, in particular the large ones, such as pLVPK, in K. pneumoniae. The reason is that ATP deficiency renders the maintenance of additional genetic elements energetically unfavorable, so they are gradually eliminated from the bacterial population [41]. On the other hand, the hypervirulence of K. pneumoniae strains may exacerbate oxidative stress, to which the pathogens per se are also exposed. The functioning of adaptation systems is mediated by HPr-dependent processes, including superoxide dismutase activation, colony formation, and biofilm development [42]. Gao et al. [42] demonstrated that ptsH in Bacillus cereus plays a pivotal role in the production of Mn-dependent superoxide dismutase, biofilm development, and surface colonization. Similar mechanisms are likely to exist in K. pneumoniae, where the PTS system may contribute to linking of the metabolic state of the cell to its capacity to retain hypervirulence plasmids under stress conditions that, in persister cells, are associated with reduced metabolic activity (Fig. 3).

The rmpA and rmpA2 genes encode regulators enhancing the production of a dense mucous capsule. This thick physical barrier effectively shields the bacterium from phagocytic uptake by immune cells, making it “invisible” to the host’s first line of defense. The iucA and iroB genes encode components of the siderophores aerobactin and salmochelin, respectively. These molecules act as molecular scavengers as they actively sequester essential iron from host proteins (transferrin and lactoferrin) and deliver it to the bacterial cell. The transporter encoded by peg-344 complements this system, thus ensuring the delivery of additional nutrients across the membrane.

The ptsH gene is a key determinant of bacterial resilience. Under stress conditions, such as antibiotic exposure or immune response, it triggers the transition of a subset of the bacterial population into the bacterial persistence state (metabolically inactive dormant cells). These persisters are invulnerable to most antibiotics, which target actively proliferating bacteria.

**Fig. 4:**
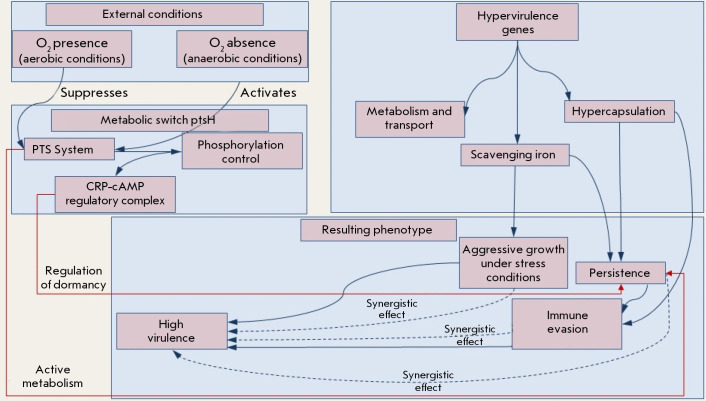
Convergent regulation of virulence and persister formation in K. pneumoniae: the role of the PTS System (ptsH) and oxygen-dependent mechanisms

Figure 4 schematically depicts the interaction between the products of hypervirulence genes and the ptsH gene in ensuring bacterial culture survival under stress conditions.

The uniqueness of the proposed scheme lies in the fact that the ptsH component of the PTS system was shown to function as an oxygen-sensitive switch. The CRP–cAMP complex is activated under anaerobic conditions, upregulating the expression of virulence genes, whereas under aerobic conditions, the system shifts toward the persistence mode to ensure longterm bacterial survival within the host. This integrative model explains the ability of K. pneumoniae to rapidly shift between active infection and persistence, which is a feature of critical importance in the development of chronic infections.

Hence, the combined activity of hypervirulence gene products enables the bacterial cell to exert pleiotropic effects that provide protection against host immune defenses, while persistence factors trigger mechanisms facilitating prolonged survival in the host organism.

A notable pattern was observed between the identified sequence types (ST) of K. pneumoniae and the number of hypervirulence genes detected. Thus, ST23, ST268, ST86, ST534, and ST219 in our cohort consistently harbored ≥ 2 hypervirulence genes (100% of cases) [39], whereas ST101 and ST395 also displayed a strong propensity for hypervirulence, with two or more hvKp genes identified in 7 out of 10 ST101-positive samples and in 10 out of 18 ST395- positive samples. Conversely, ST512, ST14, ST109, ST17, and ST491 appeared to consistently harbor fewer than two hvKp genes (100% of cases) and ST39 rarely contained any hypervirulence genes at all (only 1 out of 14 cases).

Various sequence type (ST) and capsular type (KL) combinations were identified among the K. pneumoniae isolates examined; the most frequent ones were ST39/KL23 (n = 13), ST395/KL39 (n = 9), ST512/KL107 (n = 9), ST395/KL2 (n = 9), and ST101/KL17 (n = 9). An analysis of the subgroups based on ST/KL combinations stratified by the presence or absence of ptsH revealed no statistically significant differences (Table 1). Seven ST/KL combinations were found in both the ptsHpositive and ptsH-negative groups: ST101/KL17, ST14/ KL2, ST147/KL64, ST39/KL23, ST395/KL2, ST395/ KL39, and ST512/KL107. The remaining combinations were identified as singletons, restricted to a set subgroup: 14 ptsH-positive samples had the following ST/KL combinations: ST86/KL2, ST336/ KL25, ST109/KL30, ST39/KL62, ST1805/KL48, ST29/KL63, ST1878/KL125, ST2567/KL10, ST252/KL51, ST219/KL114, ST107/KL103, ST11/KL23, ST1393/KL8, and ST23/KL1; whereas 11 ptsH-negative samples displayed the combinations ST1726/KL81, ST4041/KL30, ST377/KL102, ST4337/KL39, ST3601/KL151, ST395/KL108, ST17/KL25, ST23/KL57, ST534/KL164, ST520/KL2, and ST491/KL118. The ST and/or KL types could not be determined for the remaining ten samples.

**Table 1:** Characterization of K. pneumoniae strains based on their sequence type (ST) and capsular (K-locus, KL) type depending on the ptsH gene

Number (n = abs.)	ptsH	ST	KL	ptsH	Number (n = abs.)
4	-	ST101	KL17	+	5
1	-	ST14	KL2	+	1
3	-	ST147	KL64	+	1
8	-	ST39	KL23	+	5
4	-	ST395	KL2	+	5
3	-	ST395	KL39	+	6
6	-	ST512	KL107	+	3

A comparison of findings across different studies reveals considerable variety in the association between phenotypic and genotypic hypervirulence traits and sequence type [24, 25, 26, 27]. This may be indicative of the heterogeneity in plasmid prevalence and accumulation of plasmid-borne hypervirulence genes in strains belonging to different sequence types. Interestingly, in our study, the only strain carrying the ptsH gene in combination with all five hvKp genes – ST23 – has also been reported in other publications to exhibit phenotypic features and the fullest genetic repertoire of hypervirulence [24, 25, 26, 27]. It is quite possible that passaging of this particular strain at conditions favorable to persister cells formation may have allowed it, through several rounds of co-evolution, to acquire the enhanced invasive capacity and survivability upon antibiotic therapy across different geographic regions. It is noteworthy that the cited studies report this sequence type to be a multidrug-resistant strain. We hypothesize that the presence and transfer of the ptsH gene, as prerequisites for persister formation in K. pneumoniae, may have contributed to the emergence and stable maintenance of hvKp genes within plasmids, thereby equipping the bacterium with a “symbiotic arsenal” conferring enhanced adaptability to stressful microenvironments, including in the diverse host niches. Lim et al. have recently corroborated the essential role played by plasmid-encoded genetic virulence factors in the manifestation of the K. pneumoniae infection across various organ and tissue niches [43, 44].

## CONCLUSIONS

The present study revealed a high prevalence of hypervirulence genes in clinical K. pneumoniae isolates, reaching 64.1%. However, the full set of five “classical” hypervirulence genes was detected in fewer than 1% of the strains isolated in adult ICU patients. A significant convergence between hvKp genes and the ptsH gene encoding bacterial persister formation was identified in K. pneumoniae strains. We demonstrated for the first time that, in the presence of the full spectrum of aerobactin siderophore genes, the ptsH gene, which governs bacterial persister formation, can promote the accumulation of hypervirulence genes within the K. pneumoniae population. Hence, these findings suggest that the ptsH gene and HPr protein encoded by it may constitute an important element in the complex regulatory network ensuring the stable maintenance of plasmids carrying the hypervirulence genes in K. pneumoniae. Our findings point to the potential role played by bacterial persistence in the emergence of hypervirulent strains. Future research in this area may pave the way for developing novel therapeutic strategies targeting the PTS system and the metabolic pathways associated with it as new molecular targets for combatting infections caused by hypervirulent K. pneumoniae strains.

## References

[1] Ernst CM., Braxton JR., Rodriguez-Osorio CA. (2020). Adaptive evolution of virulence and persistence in carbapenem-resistant Klebsiella pneumoniae.. Nat Med..

[2] Haddy RI., Lee M 3rd., Sangal SP., Walbroehl GS., Hambrick CS., Sarti GM. (1989). Klebsiella pneumoniae bacteremia in the community hospital.. J Fam Pract..

[3] Andreev SS., Ketskalo MV., Narusova PO., Lysenko MA. (2023). Secondary infections in patients with extremely severe COVID-19 during ECMO.. General Reanimatology..

[4] Russo TA., Shon AS., Beanan JM. (2011). Hypervirulent K. pneumoniae secretes more and more active iron-acquisition molecules than “classical” K. pneumoniae thereby enhancing its virulence.. PLoS One..

[5] Pomakova DK., Hsiao CB., Beanan JM. (2012). Clinical and phenotypic differences between classic and hypervirulent Klebsiella pneumonia: an emerging and under-recognized pathogenic variant.. Eur J Clin Microbiol Infect Dis..

[6] Tutelyan AV., Shlykova DS., Voskanyan SL., Gaponov AM., Pisarev VM. (2022). Molecular epidemiology of hypervirulent K. pneumoniae and problems of health-care associated infections.. Bull Exp Biol Med..

[7] Mba IE., Mba TO., Uwazie CK., Aina FA., Kemisola AO., Uwazie IJ. (2025). New insights and perspectives on the virulence of hypervirulent Klebsiella pneumoniae.. Folia Microbiol (Praha)..

[8] (2024). World Health Organization. Antimicrobial Resistance, Hypervirulent Klebsiella pneumoniae - Global situation.. Disease Outbreak News..

[9] Tang Y., Du P., Du C. (2025). Genomically defined hypervirulent Klebsiella pneumoniae contributed to early-onset increased mortality.. Nat Commun..

[10] Lam MMC., Wyres KL., Judd LM. (2018). Tracking key virulence loci encoding aerobactin and salmochelin siderophore synthesis in Klebsiella pneumoniae.. Genome Med..

[11] Shon AS., Bajwa RP., Russo TA. (2013). Hypervirulent (hypermucoviscous) Klebsiella pneumoniae: a new and dangerous breed.. Virulence..

[12] Liao Y., Gong J., Yuan X., Wang X., Huang Y., Chen X. (2024). Virulence factors and carbapenem-resistance mechanisms in hypervirulent Klebsiella pneumoniae.. Infect Drug Resist..

[13] Bulger J., MacDonald U., Olson R., Beanan J., Russo TA. (2017). Metabolite transporter PEG344 is required for full virulence of hypervirulent Klebsiella pneumoniae strain hvKP1 after pulmonary but not subcutaneous challenge.. Infect Immun..

[14] Russo TA., Olson R., Fang CT. (2018). Identification of biomarkers for differentiation of hypervirulent Klebsiella pneumoniae from classical K. pneumoniae.. J Clin Microbiol..

[15] Russo TA., Alvarado CL., Davies CJ. (2024). Differentiation of hypervirulent and classical Klebsiella pneumoniae with acquired drug resistance.. mBio..

[16] Wang Q., Ye MY., Hong C., Li ZP., Lin L. (2025). The mechanisms of resistance, epidemiological characteristics, and molecular evolution of carbapenem-resistant hypervirulent Klebsiella pneumoniae.. Lab Med..

[17] Russo TA., Marr CM. (2019). Hypervirulent Klebsiella pneumoniae.. Clin Microbiol Rev..

[18] Harrison E., Brockhurst MA. (2012). Plasmid-mediated horizontal gene transfer is a coevolutionary process.. Trends Microbiol..

[19] (2021). Probing the plasmid paradox.. Nat Ecol Evol..

[20] Dewar AE., Belcher LJ., Scott TW., West SA. (2024). Genes for cooperation are not more likely to be carried by plasmids.. Proc Biol Sci..

[21] MacLean RC., Lood C., Wheatley RM. (2025). Chromosomal capture of beneficial genes drives plasmids toward ecological redundancy.. ISME J..

[22] Dewar AE., Thomas JL., Scott TW. (2021). Plasmids do not consistently stabilize cooperation across bacteria but may promote broad pathogen host-range.. Nat Ecol Evol..

[23] Hammad HA., Abdelwahab R., Browning DF., Aly SA. (2025). Genome characterization of carbapenem-resistant hypervirulent Klebsiella pneumoniae strains, carrying hybrid resistance-virulence IncHI1B/FIB plasmids, isolated from an Egyptian Pediatric ICU.. Microorganisms..

[24] McElheny CL., Iovleva A., Chen N. (2025). Prevalence and features of hypervirulent Klebsiella pneumoniae in respiratory specimens at a US hospital system.. Infect Immun..

[25] Gan L., Mao P., Tian Z. (2025). Higher prevalence of hypervirulent Klebsiella pneumoniae isolates with high-risk multidrug resistance in Asia.. J Infect Public Health..

[26] Semenova DR., Nikolaeva IV., Fialkina SV., Khaertynov KS., Anohin VA., Valiullina IR. (2020). Frequency of colonization with “hypervirulent” Klebsiella pneumoniae strains of newborns and infants with community-acquired and nosocomial klebsiella infection.. Rossiykiy Vestnik Perinatologii i Pediatrii (Russian Bulletin of Perinatology and Pediatrics)..

[27] Samoilova AA., Kraeva LA., Mikhailov NV. (2024). Genomic analysis of virulence and antibiotic resistance of Klebsiella pneumoniae strains.. Infektsiya i Immunitet (Russian Journal of Infection and Immunity)..

[28] Niu H., Gu J., Zhang Y. (2024). Bacterial persisters: molecular mechanisms and therapeutic development.. Signal Transduct Target Ther..

[29] Kalashnikova TP., Arsenyeva YA., Kamenshchikov NO. (2024). Antibacterial effect of nitric oxide on causative agents of hospital-acquired pneumonia (experimental study).. General Reanimatology..

[30] Eremenko AA., Marchenko TV., Nikoda VV., Zokoev AK., Skripalenko DA. (2023). Endotoxin and cytokines removal with adsorption device in a child with sepsis after transplantectomy (case report).. General Reanimatology..

[31] Demkina EV., Loiko NG., Mulyukin AL. (2015). Effect of inherent immunity factors of development of antibiotic tolerance and survival of bacterial populations under antibiotic attack.. Microbiology..

[32] Tutelyan AV., Gaponov AM., Pisarev VM., El-Registan GI. (2015). Microbial dormancy and prevention of healthcare-associated infections.. Ter Arkh..

[33] Wang X., Ma W., Shan J. (2023). The phosphotransferase system gene ptsH affects persister formation in Klebsiella pneumoniae by regulating cyclic adenosine monophosphate levels.. Int J Antimicrob Agents..

[34] Prjibelski A., Antipov D., Meleshko D., Lapidus A., Korobeynikov A. (2020). Using SPAdes de novo assembler.. Curr Protoc Bioinformatics..

[35] Shelenkov A., Mikhaylova Y., Yanushevich Y. (2020). Molecular typing, characterization of antimicrobial resistance, virulence profiling and analysis of whole-genome sequence of clinical Klebsiella pneumoniae isolates.. Antibiotics (Basel)..

[36] Shelenkov A., Petrova L., Zamyatin M. (2021). Diversity of international high-risk clones of Acinetobacter baumannii revealed in a Russian multidisciplinary medical center during 2017-2019.. Antibiotics (Basel)..

[37] (2025). Institut Pasteur. Klebsiella pneumoniae species complex. Institut Pasteur MLST databases and software.. https://bigsdb.pasteur.fr/klebsiella/.

[38] (2025). Center for Genomic Epidemiology. ResFinder. DTU Food..

[39] (2025). NHC Key Laboratory of Systems Biology of Pathogens. Virulence Factor Database (VFDB)..

[40] Robertson J., Bessonov K., Schonfeld J., Nash JHE. (2020). Universal whole-sequence-based plasmid typing and its utility to prediction of host range and epidemiological surveillance.. Microb Genom..

[41] Ah-Seng Y., Rech J., Lane D., Bouet JY. (2013). Defining the role of ATP hydrolysis in mitotic segregation of bacterial plasmids.. PLoS Genet..

[42] Gao T., Ding M., Yang CH., Fan H., Chai Y., Li Y. (2019). The phosphotransferase system gene ptsH plays an important role in MnSOD production, biofilm formation, swarming motility, and root colonization in Bacillus cereus 905.. Res Microbiol..

[43] Lim C., Zhang CY., Cheam G. (2025). Essentiality of the virulence plasmid-encoded factors in disease pathogenesis of the major lineage of hypervirulent Klebsiella pneumoniae varies in different infection niches.. EBioMedicine..

[44] Gong L., Wang X., Zheng B. (2025). Context-dependent virulence in Klebsiella pneumoniae: deciphering niche-specific adaptation and virulence-resistance interplay.. EBioMedicine..

